# Notes and Miscellany

**Published:** 1911-08

**Authors:** 


### Notes and Miscellany.

TO THE PROFESSION:

Any physician receiving circulars advertising a book published by one W. J. Jackman and purporting to contain an introductory chapter by me, will confer a favor by sending such circulars with the envelope in which they were received to me.

G. FRANK LYDSTON, M. D., Chicago, Ill.

---

PRIZE ESSAY.—Under the auspices of the American Society for the Study of Alcohol and Other Narcotics, Dr. L. D. Mason of Brooklyn, N. Y., vice-president of the society, offers a prize of $150 for the best essay on the following topic: *"The Biological and Physiological Relations of Alcohol to Life."* The essay must be the result of original research, which shall confirm or disprove the present theories of the inherited effects of alcoholic degeneration and indicate how far the defects of the parents are transmitted to the children. Such work may be carried on in man or animal, and the results may be illustrated by drawings or photographs, and must be typewritten and sent to the office of the secretary before July 1, 1913.

This offer is open to students in all countries, and each essay should be accompanied by a motto and a sealed envelope containing the same, with the author's name and address.

A committee of award, of which Dr. W. S. Hall, professor of physiology in the Northwestern University, Chicago, Ill., is the chairman.

All inquiries should be addressed to Dr. T. D. Crothers, Secretary, Hartford, Conn.

---

Dr. Luther Halsey Gulick, director in the Russell Sage Foundation, and formerly director of physical training in the New York public schools, has written a noteworthy series of articles on athletics in their relation to health, for *Lippincott's Magazine.* The first of these, "The Requirements of Healthful Exercise," appeared in the June number. That in July is entitled "Games and Gangs." It deals with the ever-present boy problem, and will prove a revelation to worried parents and harassed teachers. Dr. Gulick's long and varied career in the fields of hygiene and education renders him well equipped for writing on such topics, and the papers may be regarded as authoritative.

---

To Shave Without Soap, Lather, Brush or Water.—I am using with delight, *Daniel's Texana,* a shaving cream which converts shaving from a bore to a luxury. It is absurd to say that any soap lather or warm water or anything else "softens the beard." A hair is like a cane or straw. Its cortex is impervious. Nothing "softens" it. The cream lubricates it and enables the blade to take hold, just as a drop of oil on the point of the drill facilitates the process of drilling into a piece of iron or steel. The process of shaving consists of lubricating the beard,, rubbing it in with the hand; then scrape it off with a sharp razor and wipe the face with a dry towel and the thing is done. It requires no washing of the face, which is left soft and smooth, and the razor is not gummed up as with lather, and can be wiped off. I am delighted with it. I shave in two minutes.

